# Identification of environmentally relevant chemicals in bibliographic databases: a comparative analysis

**DOI:** 10.1186/2193-1801-2-255

**Published:** 2013-06-05

**Authors:** Ole Ellegaard, Johan A Wallin

**Affiliations:** University Library, University of Southern Denmark, Campusvej 55, Odense, 5230-M Denmark

**Keywords:** Chemical substances, CAS-numbers, Chemical databases, Indexing methods, Retrieval methods

## Abstract

Valid and reliable information on the use and effects of chemicals is a key factor in the industry and not least within many regulatory agencies. Identification data from lists of substances sometimes leads to incomplete bibliographic analysis in the major chemical databases. The present study takes as its starting point environmentally important chemicals and the retrieval of selectively chosen substances in the four databases: SciFinder, Web of Science (WoS), Scopus and Google Scholar. The way chemical data are stored in the databases plays a major role in the recovery process but differences in coverage, sometimes major, are still found. No single database records all publications about a substance. Inspection of individual titles is necessary when performing a complete count of references. Special care is taken in order to make data from the different databases comparable using the same journals and time periods (2000-2009). A number of nomenclature as well as problems related to the chemical structure and function, often inherent in quantitative or qualitative bibliographic studies of chemicals, are discussed. The practical implications for registration of chemicals in different databases are demonstrated.

## Background

During a recent study of the Matthew effect in research on chemicals in environmental studies (Grandjean et al. 2011), we identified a problem in properly identifying the chemicals in the major bibliographic scientific databases Chemical Abstract Service (SciFinder) and Web of Science (WoS). The main purpose of the study was to investigate whether inertia could be documented for environmental research on upcoming chemicals. We needed an exhaustive registration of the chemicals in the bibliographic databases in order to assess the amount of research conducted on the chemicals.

As the amount of information, or more precisely published information, grows at an ever increasing rate, it is of course important to extract the most relevant publications with regard to the subject at hand. This situation is encountered within all scientific areas, not only environmental studies. A first stage in any risk assessment is normally based on a literature review (University of Cambridge 2011). Pitfalls, whereby information on the effects of chemicals is missed, are very important to identify when we deal with environmental relevant chemicals (Hall & Walton 2004). It is even asserted that data gaps, due to companies not providing sufficient information, threaten chemical safety laws throughout Europe (Gilbert 2011).

The extraction of information about chemical substances has been studied for a number of years within the field of Chemoinformatics (Leach & Gillet 2007). Database content is often a mixture of factual and bibliographic content as in, for example, the major chemical database SciFinder which in its present form includes chemical data (Guerbet and Guyodo 2002). A number of studies have focused on the available commercial or free data sources. Most work deals with content analysis and advantages or disadvantages in the use of the individual databases (Patterson et al. 2002; Ludl et al. 1996; Frandsen & Nicolaisen 2008). In the present study we will take the bibliometric approach. We will focus on the methods the most important chemical databases apply in indexing of chemicals. The important part of chemical information management is not only about storing the information in databases- retrieval and evaluation has to follow (Voigt & Welzl 2002).

A comprehensive literature study of the effect of chemicals must meet two main criteria: 1. A sufficient number of bibliographic databases must be included in order to cover the vast amount of published information about the substances (Hood & Wilson 2001). 2. The indexing of the databases and the information retrieval must ensure that all relevant literature is extracted.

Ideally, a chemical is properly identified through the use of a Chemical Abstract Service number (CAS-number or Registry number) which is far more convenient than dealing with the full chemical IUPAC (International Union of Pure and Applied Chemistry) -name or CA-index name). The CAS number designates a unique chemical substance and is extremely useful for scientific and technical communication. For example, United States Environmental Protection Agency (USEPA) relies on CAS numbers for the definite identification of chemical substances (EPA-United States Environmental Protection Agency 1998). Also in the European Union (EU), the REACH (Regulation on Registration, Evaluation, Authorization and Restriction of Chemicals) candidate list relies on a proper identification of these problematic substances (REACH 2007). Here we meet the first obstacle because WoS do not register CAS numbers in a proper index. Registration only takes place when the numbers are present in the title or abstract of the publications. We assume that chemical substance identification is more carefully treated by CAS. Obviously, this is the case through the unique identification by CAS numbers. However, in performing a practical analysis a number of problems showed up which may distort the results obtained.

The major general problems pertinent to chemical substances can be identified and categorized as follows:

1. Different names of substances. Are chemical names assigned as keywords?
2. Different trivial names or part of product names.
3. Use of abbreviations in names.
4. Different indexing policies for CAS numbers.
5. Change of CAS numbers or multiple numbers.
6. Chemical name is only a fractional part of a more complete name. As an example cis-trans isomers can have different CAS numbers. Different stereoisomers of a substance may also have different CAS numbers while the same substance with unspecified stereochemistry has its own number. Tautomerism also leads to the existence of interconvertible forms of substances.
7. Classes of substances e.g. polymers are often not registered in an unambiguous manner. CAS numbers may exist for branched or cross-linked polymer molecules of an unknown composition. Actually, no such thing as a ‘pure’ polymer exist (Peacock and Allison 2006).
  Substances may also form part of a biological material, salts, mixtures, hydrates or alloys with its own registry numbers. Finally, substances which are ionized or radicals may pose special problems.
8. Overall different indexing policy for scientific work in articles.

With regard to option 8, this is part of a more comprehensive range of obstacles to a consistent registration in the literature. Does the database include patents, editorials or errata? Does it include ‘grey’ literature such as reports, congress contributions, thesis etc.? The present work only partly deals with these issues. Further problems can be due to the different number of periodicals which are included in the databases and the time period covered. It is well known that journals from third world countries and articles published in a language different from English are not so well represented in the large bibliographic databases (Neuhaus & Daniel 2008). This will skew the result of a statistical analysis due to the vast increase in the literature from some of these countries. This problem is increasing because in the later years there has been a surge in the number of publications from countries such as China and India with an expanding scientific sector. Finally, a periodical can change name, continue under a different name or split up under different names.

The above mentioned points obviously relate to the major chemical databases, e.g. SciFinder, Web of Science (WoS) or Scopus, but poses problems for any bibliographic database which indexes the chemical literature.

All these points make it difficult to quantify the use of chemicals as documented from the frequency of publications about the substances in the literature. Research on certain chemicals may not be properly documented in the literature or the various methods of analysis may fail to take all the works into account. This can lead to erroneous conclusions on the relative impact of chemicals and their use or misuse in the society.

We investigate the major bibliographic databases Web of Science (WoS), SciFinder (Chemical Abstract Service), Scopus and an example of a resource with open access: the web search engine Google Scholar (hereafter designated as ‘Scholar’).

The scope of these databases has been discussed elsewhere (Li et al. 2010). We only briefly notice that all bases include general coverage of the natural sciences and medicine while SciFinder has, in addition, a special good coverage in chemistry. Regarding the types of publications, all databases analyze journals and conference proceedings. Patents are also a very import source of information about chemical substances. SciFinder is a well-known source to the patent literature and presents the possibility of extending substance searching via Markus-structures. Scholar also covers patents in a more simplified manner through ‘free-text’ searches. WoS and Scopus do not index patents. The scope of Scholar also includes more ‘grey’ material from professional societies, online repositories and web sites. Scholar provides no information about the period indexed but all databases cover the period 2000–2009 included in the present study. Overall, Scholar presents the poorest documentation of their working procedures and indexing policies (Frandsen & Nicolaisen 2008), which makes elementary bibliometric analysis difficult to perform (Neuhaus & Daniel 2008).

A major difference exists in principle between the ways SciFinder, Scopus and WoS deal with chemical data. In SciFinder chemical names are translated to CA-index names and CAS numbers (Ridley 2009). WoS uses the chemical name as found in the article either in the title, abstract or keyword registers. Scopus also includes CAS- numbers as well as MeSH and EMTREE drug terms in keywords but otherwise treats chemicals in the same way as WoS. Substances often change CAS number during their registration at CAS. Often, one cannot rely on a single number to retrieve all information about a substance. A CAS number can be given to a substance with a trade name but without any structural information associated. The CAS number can be deleted if the substance is later related to a known substance. Alternate registration numbers are used when different structure representations of the same compound exist. Normally, it does not pose a special problem because all variants of a given CAS number are connected during the search process. On the other hand, stereo- or other types of isomers with different physical or chemical properties may be given separate CAS numbers which are not connected during the search process. As the only database considered here, SciFinder offers structure searches. It sometimes proves to be the best method for uncovering different stereoisomers or isotopic substituted substances. In case of classes of compounds which are unspecified or with unknown/variable composition, the publications are not always found directly from the CAS number. Examples include the quaternary ammonium compounds such as alkylbenzyldimethylammonium chlorides (61789–72) listed by the Environmental Protection Agency (EPA-United States Environmental Protection Agency 1998). No publications are indexed under this CAS number in SciFinder (Grandjean et al. 2011).

The SciFinder approach clearly has the advantage of being the most unambiguous. On the other hand, a selective approach is introduced because not all chemicals are catalogued during the registration process. It can be due to the fact that 1. The journal under registration does not apply CAS numbers or 2. The name of a chemical is not translated into a proper CAS number.The first issue is encountered in a number of SciFinder searches which include the Medline database. This database includes a large number of important medical journals which do not incorporate these numbers. Co-searching of Medline is the default standard for SciFinder searches and in many situations leads to duplicate publications. These publications are filtered out in the search results, although one should be aware, that no duplicate removal takes place when combining the search results. Also, a slight difference in the bibliographic data of the publications may result in an erroneous removal of duplicates. This is a situation often encountered when dealing with citation data. Finally, indexing practices in the two databases may lead to additional publications not found in the other database. Certain isomers with different CAS numbers in the CAPLUS database have e.g. the same number in Medline (Ridley 2009). In the present work, due to the above mentioned risk of duplicate publications, we include and analyze only publications from the CAPLUS database.

With regard to option 2, not all chemicals, or more precisely their description, in the articles seems to warrant an inclusion in the database. Often, the introduction section of many research articles summarizes only previously known information (Ridley 2009). Long lists of substances can be neglected if the author’s main focus is on the description of properties of the substances. A chemical can be noted as a reagent, intermediate or otherwise, only mentioned in an inferior context with regard to the main purpose of the article. As an example, reagents are not indexed unless they are new or used in a novel way (Chemical Abstract 2002). Of course, this may work the other way round. Chemicals are often included with CAS number in the database without any priority range. In the latter case roles can be associated with the chemicals and in this way facilitate the search process.

The same problem is also encountered in WoS. The data base does not register CAS numbers at all unless they are mentioned in the title or abstract fields. Identification of chemicals in the WoS database is then completely dependent on a proper selection of chemical names by the pertinent authors. The later introduced keyword and keyword-plus fields do not apply systematic registration of chemicals either. In Scopus, with few exceptions, a one-to-one correspondence between chemical name and CAS number seems to exist. Obviously, it does not allow the same differentiation between isomers and use of trivial names as observed in SciFinder.

The databases do not always make a clear distinction between different forms of chemicals e.g. acids and salts of these. Examples are sulfonic acids and sulfonates. Organic bases and hydrochlorides are often intermixed during the registration process- an example is lidocaine (137-58-6) and lidocaine- hydrochloride (73-78-9). Unspecified compounds pose special problems e.g. chlorphenol (25167-80-0) is in unspecified compound of phenol (108-95-2) and chlorbenzene (108-90-7) with its own publications in SciFinder.

Finally, Scholar seems to harvest all data from the publications list of cited references and, to an unknown degree, the full text into a bibliographic record. The former in particular may introduce a lot of inferior bibliographic records with no obvious connection between search term and the content of the record. These records can only be found by a manual inspection of the list of publications. The advantage of the Google approach seems to be a more simple indexing practice based on automatic algorithms. The disadvantage is the ‘noise’ from more or less irrelevant search results and even duplicates. The latter could emerge from articles included in genuine journals as well as institutional repositories. This may pose a particular problem for the statistical, bibliometric analysis in this database where search results are not individually judged for relevance.

## Method

The main aim of the present work is to document and discuss the pitfalls in performing analysis of chemical substances and the frequency of their existence in publications in the scientific literature. The results obtained on the registration of chemicals may in some cases lead to apparently deviating results. In all cases we try to explain the results within the context of the points raised above. A number of cases studied with different representative chemicals in different journals and databases will be presented. The main purpose is to illustrate and discuss the practical consequences of the variety of indexing methods.

Certain chemical substances are selected from a so called POP-list of Persistent Organic Pollutants published under United Nations Environment Programme (UNEP- United Nations Environment Programme 2008). These chemicals are in special need of documentation as they pose a special risk of adverse effects to human health and the environment. These chemicals are part of ‘the dirty dozen’ named by the Intergovernmental Forum on Chemical Safety (WHO’s World Health Organization official site IFCS 2004). We also select a number of well-known chemicals which are often recognized under different trade names. The latter type of chemicals may pose special problems as they are documented under these different names in the literature. In order to assess the documentation of chemicals in the literature we can, of course, only perform spot tests with few chemicals. We will not provide, with the present investigation, an overall quantitative measure of the defective identification of chemicals. Instead, we investigate whether the same search profile lads to different results in four major different databases used by the scientific community.

SciFinder apply an intelligent search interface: ‘research topic(rt)’. This interface includes ‘behind the scene’ alternative spellings, plurals and CAS numbers of substances as well as a weighting algorithm and can be very useful. The actual search terms are treated by SciFinder either ‘as entered’ or as ‘a concept’. We apply the latter in almost all cases because it leads to the most unambiguous results with the largest number of publications.

We base our analysis on a straight comparison regarding the number of indexed publications within the same database as well as between different databases. The document types indexed can be different. We apply the term ‘article’ in case of genuine journal articles while the broader term ‘publication’ includes e.g. patents, reports and dissertations as well.

The substances are counted without regard for their role (e.g. analysis, synthesis or technical use) in the publications. A number of periodicals within the subject area of Environmental Science are extracted and applied. We use the exact same periodicals and periods for the analysis which will guarantee that no artifacts show up in the results.

We chose the time period 2000–2009 in our analysis. The latest couple of years were not selected because the registration process can lag somewhat behind the publication process. On the other hand, we must ensure that the registration method did not change during the period. As an example, WoS introduced ‘keyword’ registration as well as abstracts in 1990. These additions could significantly improve the ‘hit-rate’ with regard to WoS searches for articles published after that year.

Due to these facts, we picked a few journals in order to perform a more individual analysis. The actual articles dealing with a certain substance were compared for the four different data bases. This method gives a more complete overview of the total amount of publications with reference to the individual substance as well as the relative number of unregistered publications within the bibliographies. This painstaking procedure may further reveal the practical implications of the eight points mentioned above.

The restriction to environmental journals is rather arbitrarily chosen with respect to the main objective of our investigation. We do not believe the results will be significantly different within other subject areas. Finally, the comparison between the numbers of publications in the following is based on the stated search terms either using the most common chemical names or registry numbers. The possibility exists, of course, that additional chemical synonyms could lead to a few more publications.

## Results

### Environmentally important chemicals

In Table 1, we consider different chemicals from the European Environmental Agency list as well as a few polymers and their occurrence in different periodicals during the period 2000–2009. We found a relatively large discrepancy between the registration in WoS and SciFinder, although fair agreements are found for some chemicals. In a minority of instances the largest numbers of articles are found in WoS. In the case of tributyltin 50 percent more articles in the journal Chemosphere are counted in WoS compared to SciFinder. Actually, this surplus of more than twenty articles in WoS is instead indexed under the name tributyltin hydride (699-73-3) in SciFinder. In this way a superficial search of the CAS number of tributyltin in SciFinder fails to produce some of the articles found in WoS. Also of note is the major difference we see in the case of flouranthene. The compound passes the indexing policy of SciFinder almost three times more often than observed in WoS.

**Table 1:** **Different environmentally important chemicals**^**1)**^**and their occurrence (in number of articles**) **in selected periodicals as registered by SciFinder and WoS between the years 2000**-**2009**

Substance	CAS nr	Journal	WoS/tp	SciFinder/rn
MTBE or methyl-tert-butyl-ether	1634-04-4	Environmental Science and Technology	113	141
Benzene	71-43-2	Environmental Science and Technology	281	375
TBT or tributyltin	36643-28-4	Chemosphere	63	42^2)^
Bisphenol A	80-05-7	Chemosphere	124	104
Mercury or Hg	7439-97-6	Environmental. Science and Technology	590^4)^	489
DES or diethylstillbestrol	56-53-1	Environmental Toxicology and Chemistry	7	15
DBCP or 1,2 dibromo-3-chloropropane	96-12-8	International Journal of Environmental Science and Technology	3	3
PCE or perchlorethylene^3)^	127-18-4	Environmental Science and Technology	149^4)^	242
Fluoranthene	206-44-0	Environmental Toxicology and Chemistry	81	214
Pyrene	129-00-0	Chemosphere	219	345
Polypropylene^5)^	9003-07-0	Polymer Degradation and Stability	504	192
Polystyrene	9003-53-6	Environmental Science and Technology	51	34

tp=topic, rn=registry number, nr=number.

*1)* From: ‘European Environmental Agency (2001) *Late lessons from early warnings*: *the precautionary principle 1896*-*2000*, Copenhagen’ and the polymers: polypropylene and polystyrene.

*2)* Includes the chloride (1461-22-9).

*3)* Also tetrachlorethylene and PCE.

*4)* May include some false hits as the abbreviations have other meanings.

*5)* Also polypropene.

The table also demonstrates a rather annoying problem when dealing with literature studies of chemicals. The widespread use of abbreviations in chemical names may lead to false hits. An example is the use of DES for the synthetic estrogen diethylstillbestrol. This term has many alternative meanings in a similar context, such as DES-gene or Dysequlibrium Syndrome. Of course, this problem is most pronounced in title- or free text-searches but may be less prevalent in databases with a practice of chemical indexing such as SciFinder.

The data further illustrates that the search of polymers is difficult to perform when we compare SciFinder and WoS. A simple CAS number search of polypropylene in the journal ‘Polymer Degradation and Stability’ leads to fewer articles than the chemical name search in WoS. If we combine with the chemical names in SciFinder, the number of hits increases to 332 which still is far less than obtained in WoS. The reason is mainly that polypropylene can be registered in SciFinder as isotactic-polypropylene, a copolymer or a blend. In case of the polymer polystyrene, the SciFinder CAS number search also produces fewer hits in the journal ‘Environmental Science and Technology’ compared to the result in WoS. If we add the results from the chemical name search in Scifinder, we obtain 63 hits which are larger compared to WoS in this particular case.

### POP chemicals

Table 2 shows the occurrence of chemicals from the Stockholm list of Persistent Organic Chemicals in the journal Chemosphere as registered in SciFinder, WoS, Scopus and Scholar during 2000–2009. The registration in SciFinder is almost independent of the search strategy. Search by ‘research topic’ gives a small but consistently larger number of articles than the search by CAS number. Only for the compound chlordane is there a major difference. In the case of WoS far fewer hits are found for all six compounds. A Scopus search in the ti, ab and kw indexes produces fewer hits than SciFinder and is more in line with WoS although the number in most cases is slightly larger. The results for Scopus depend somewhat on the actual indexes searched. Applying the ti, ab and kw indexes produces, in almost all cases, the largest number of hits (Table 3).

**Table 2:** **Articles about chemicals from the Stockholm list of Persistent Organic Chemicals** (**POP**-**list**) **in the journal Chemosphere as registered by SciFinder**, **WoS**, **Scopus and Google Scholar**

Substance	CAS nr	SciFinder/rt	SciFinder	Scopus	WoS	Google	Google
			/rn	/ti,ab,kw	/tp ^3)^	Scholar, tot	Scholar, -cit
Aldrin	309-00-2 ^1)^	88	88	45	36	167	142
Chlordane	57-74-9 ^2)^	121	1	71	67	241	184
Dieldrin	60-57-1	114	114	62	52	236	172
Endrin	72-20-8	74	72	34	26	126	107
Heptachlor	76-44-8	111	90	57	46	183	153
Hexachlor-benzene or HCB	118-74-1	259	233	129	174	589	401

Year 2000-2009.

rt=reseach topic, ti=title, ab=abstract, kw=keyword, au=author, tot=total, cit= citations.

*1)* Found under different CAS numbers which all translates to 309-00-2.

*2)* CAS number from official list. May be registered as cis- or trans-chlordane with different number.

*3)* Topic is equivalent to the search fields ti,au,ab and kw but not keywordplus.

**Table 3:** **Number of articles in the journal Chemosphere about the POP**-**substances analyzed by different indexes in Scopus**

Substance	Scopus/ti,ab,kw	Scopus/cn	Scopus/rn
Aldrin	45	39	38
Chlordane	71	51	51
Dieldrin	62	53	53
Endrin	34	28	28
Heptachlor	57	44	29
Hexachlorbenzene	129	136	136
or HCB			

Scholar systematically leads to a larger number of articles for all compounds even if we subtract those articles where the chemical is only mentioned in the reference list. As an example, aldrin is indexed 167 times but it only represents 142 genuine articles about aldrin. The number of articles in Scholar is generally 35%-60% above the numbers in SciFinder for these POP-list chemicals. This is mainly due to the indexing of an unknown part of the full text.

The very few hits for the chlordane search with the CAS number deserve further analysis (Table 4). The term chlordane with CAS number: 54-74-9 apparently has a somewhat different meaning in SciFinder. It is listed for a compound with unspecified stereochemistry. This number is only registered once by CAS for the articles abstracted from the journal Chemosphere during 2000–2009. In the CAS registry index chlordane is an unspecified product named as ‘technical chlordane’ with CAS number: 12789-03-6 and 39 articles. Actually, searching chlordane as a ‘chemical name’ leads to ‘technical chlordane’. Instead chlordane seems to be partly registered under cis- and trans-chlordane. If we search cis-chlordane (5103-71-9) under its registration number, 74 articles are obtained while trans-chlordane gives 69 articles. Combined, the two isomers contribute 79 articles in total. If we further combine with chlordane as ‘research topic’ (121 articles, Table 4) we obtain 122 different articles. Only one additional article is added when we include the proper CAS numbers for the cis- and trans-isomers.

**Table 4:** **Number of articles in Chemosphere about the POP-substance chlordane analyzed by different CAS numbers in SciFinder and Scopus**

CAS number or name	SciFinder	Scopus
57-74-9	1	51
5103-71-9 Cis-chlordane	74	0
17436-70-3 Trans-chlordane	69	0
12789-03-6 Unspecified ‘technical’ chlordane^1)^	39	56
Chlordane/rt	121	51
**All**	**123**	**56**

Deleted CAS numbers are included for trans-chlordane.

*1.* Equivalent to chlordane/cn (cn=chemical name).

Finally, we also combine with the CAS number for ‘technical chlordane’ and all terms combined gives the total result of 123 articles in Chemosphere. Scopus, on the other hand, does not distinguish between chlordane as a chemical name and the registry number. The registry number search for ‘technical chlordane’ only leads to a slightly larger result. All three entries combined lead to almost the same number of articles. These data are also summarized in Table 4.

### Aldrin

In Table 5 we take a closer look at the substance aldrin which is one of the chemicals from the Stockholm POP list. A complete search for aldrin as a ‘research topic’ in SciFinder during the period 2000–2009 produces 2146 hits. If we use a registry number or chemical name search 2061 hits are found. All these 2061 hits are included in the ‘research topic’ search. Apparently, 85 publications are only found if we search aldrin as ‘research topic’. Overall, as much as 4% of the publications involving aldrin can be missed depending on the search procedure. We also perform an exact structure search on aldrin in SciFinder and obtain 2074 hits. In this way publications are found on aldrin irrespective of any stereo match. Further publications with isotopic substitution in aldrin are found as well. If we combine the three different methods a total of 2159 different publications could be obtained in SciFinder. 67, or less than 3%, of these publications are patents. The number of publications found in WoS is significantly smaller. Most likely, the difference is due to the more thorough indexing practice of SciFinder.

**Table 5:** **The total number of publications about Aldrin** (**309**-**00**-**2**) **registered in SciFinder and WoS during the years 2000-2009**

Database	Total count
SciFinder/rt	2146
SciFinder/rn	2061
SciFinder/str	2074
SciFinder/rt+rn+str	2159
Wos/tp	417

In Table 6, we consider the environmental journals with most articles on aldrin. The number of articles found in SciFinder is again almost the same irrespective of the search method. There is a small systematic trend for a majority of the journals that searching for the chemical as a ‘Research topic’ produces the largest number of hits. This indicates that a full connection is not always established between trivial name and CAS number in the database.

**Table 6:** **Articles in different environmental journals about Aldrin** (**309**-**00**-**2**) **as registered by WoS or SciFinder**

Journal	SciFinder	SciFinder	WoS
	309-00-2/rn	Aldrin/rt	Aldrin/tp
Chemosphere	88	88	36
Science of the Total Environment	26	31	17
Environmental Monitoring and Assessment	34	34	12
Environmental Pollution	20	21	12
Marine Pollution Bulletin	42	44	10
Environmental Science and Technology	44	44	9
Fresenius Environmental Bulletin	17	19	8
Journal of Environmental Science and Health Part B	21	21	8
Bulletin of Environmental Contamination and Toxicology	45	46	7
Environment International	14	16	7

Aldrin has a number of alternative trivial names. In SciFinder, these names searched as a ‘research topic’ produce in all cases the same number of hits as the chemical name search on aldrin itself. The lesser used trivial names are directly translated to the registry number in the database. This seems to be the general procedure with the variety of trivial names and works very well in SciFinder. The number of articles registered by WoS seems to be significantly smaller in all cases (Tables 5, 6). If we apply the many different trivial names for Aldrin mentioned above, it produces no futher hits in WoS. This demonstrates the value of the name concordance in the SciFinder database.

### Glyphosate

The data for the well-studied chemical glyphosate are shown in the Tables 7,8,9 and 10. This chemical is the most important constituent of the herbicide ‘RoundUp’. In the literature the chemical is mostly registered or indexed under the trivial name glyphosate rather than under the chemical name N-(phosphonomethyl)glycine or its many trade names. Glyphosate is registered most often in Scholar, somewhat less often in SciFinder and even less in Scopus and WoS. The numbers found in SciFinder include results with both the name glyphosate as well as its CAS number and derived from this number also the alternative trade names. If we include ‘RoundUp’ about 25% more articles are added. Almost half of these are already included under the glyphosate CAS number (Table 7). In SciFinder no less than 2849 or 45% of the publications are patents. The number of non-patent publications becomes almost similar for the SciFinder, WoS and Scopus databases. The latter has the largest number of publications most likely due to the larger number of journals indexed.

**Table 7:** **The total number of publications about Glyphosate** (**1071**-**83**-**6**) **registered in SciFinder**, **WoS and Scopus**

Database	Glyphosate	RoundUp	Total
SciFinder /rt	5779	1663	6269
WoS/tp	2995	763	3412
Scopus /ti,ab,kw	2818	1750	4250

Search terms in all cases are ‘glyphosate’ or ‘RoundUp’ in the respective indexes.

**Table 8:** **Articles about Glyphosate** (**1071**-**83**-**6**) **in a number of environmental journals as registered by SciFinder**, **WoS and Scopus**

Journal	SciFinder	WoS/tp	Scopus	Google Scholar, tot	Google Scholar, -cit
	/rt		/ti,ab,kw		
Chemosphere	34	31	28	96	47
Bulletin of Environmental Contamination and Technology	25	19	23	58	36
Clinical Toxicology	6	n.i.	9	46	27
Environmental Pollution, Amsterdam+Oxford	12	11	11	86	64
Journal of Toxicology and Environmental Health A	14	13	11	40	24
Journal of Environmental Science and Health B	14	16	13	47	33
Journal of Environmental Quality	23	22	19	75	67
Toxicology Letters	5	9	5	16	12
Environmental Toxicology and Chemistry	30	20	22	80	58
Food Additives and Contaminants	6	6	6	15	12
**Total**	**169**	**148**	**147**	**559**	**380**

n.i. = not included.

**Table 9:** **Articles about Glyphosate** (**1071**-**83**-**6**) **in the periodicals** ‘**Toxicology Letters**’ **and** ‘**Environmental Toxicology and Chemistry**’ **as registered by a different combination of databases**

Journal	Sci	WoS	Sch	WoS+Sch	Sci+Sch	Sco+Sch	Sci+WoS	Sci+So	Sci+WoS
							+Sch	+Sch	+Sco+Sch
Toxicology Letters	0	1	4	3	0	0	0	0	5
Environmental Toxicology and Chemistry	1	0	25	2	8	2	1	3	17

Sci=SciFinder, WoS=Web of Science, Sch=Google Scholar, Sco=Scopus. WoS+Sch etc. = Articles are only registered in these databases.

Entries with Google Scholar do not include articles with the word ‘glyphosate’ or ‘RoundUp’ mentioned only in the list of references.

**Table 10:** **Total count of articles about Glyphosate** (**1071**-**83**-**6**) **in** ‘**Toxicology Letters**’ **and** ‘**Environmental Toxicology and Chemistry**’ **as registered in different databases**

Journal	Sci	WoS	Sco	Sch	Total for all four
Toxicology Letters	5	9	5	12	13
Environmental Toxicology and Chemistry	30	20	22	58	59

Next, we select the ten environmental journals with the most articles in WoS or SciFinder and also include data for Scopus and Scholar (Table 8). First, if we neglect Scholar, the total number of articles in the ten journals is almost equal. If we consider all ten journals, the total difference in numbers is 22 articles or 14% more articles in SciFinder than Scopus. In contrast to the POP-list, Table 2, we find a slightly larger number of articles registered by WoS compared to Scopus. As seen in the table, three more articles are registered in the ‘Journal of Environmental Science and Health B’. WoS apply the term Glyphosate as keyword term in these articles while this is not the case in Scopus.

If we go further and look at the individual titles (Tables 9, 10), the difference in registration becomes more prominent. When we search ‘Environmental Toxicology and Chemistry’ only 18 out of 34 articles (53%) are indexed in both SciFinder and WoS. The same figure in the cases of SciFinder and Scopus is 20 out of 34 articles (59%). The numbers of articles obtained from Scholar are significantly larger with a total of 80 counts. In 22 cases the word ‘glyphosate’ is found only in the title of articles cited in the reference list, leaving 58 articles. Of these a surprisingly large number are indexed only in Scholar (25 out of a total of 59 articles or 42%).

The same pattern is seen if we look at the articles indexed in Toxicology Letters. SciFinder indexes fewer articles with the term ‘glyphosate’ than both WoS and Scholar. This does not mean that the articles indexed in WoS and Scholar is not indexed in SciFinder at all- but apparently not under the term ‘glyphosate’. The additional articles in WoS compared to Scifinder are related to meeting abstracts not indexed by the latter. The total Scholar count for ‘glyphosate’ in Toxicology Letter is actually 16 but again 4 of these are citations. This leaves only twelve articles, of which four are unique, registered only by Scholar. The very good coverage of Scholar is most remarkable, despite its simple search interface: almost all publications found in SciFinder, WoS or Scopus are also indexed in Scholar. The difference between Scholar and the other databases is mainly due to the full text indexing practice of the former.

### PFOS or perfluorooctane sulfonate

In Tables 11, 12, 13 and 14 we consider the substance PFOS which is already forbidden by the Stockholm convention, annex B on persistent organic pollutants (UNEP- United Nations Environment Programme 2008). This substance seems mainly to be registered in the literature either under its acronym PFOS, perfluorooctane sulfonate (45298-90-6) or perfluorooctane sulfonic acid (1763-23-1). In a few cases the composition of these names may lead to deviating results.

**Table 11:** **Registration of PFOS or perfluorooctane sulfonate** (**45298**-**90**-**6**) **or perfluorooctane sulfonic acid** (**1763**-**23**-**1**) **in SciFinder analyzed by related CAS**-**numbers during the years 2000-2009**

**Research topic PFOS: ’as entered’**		**Research topic PFOS: ‘as concept’*not*’as entered’**	
**848 publications**		**1247 publications**	
CAS-nr	Publications	CAS-nr	Publications
1763-23-1 (PFOS, acid)	459	335-67-1 (PFOA, acid)	224
335-67-1 (PFOA, acid)	405	45285-51-6 (PFO, ion)	34
45298-90-6 (PFOS, sulfonate)	197	1763-23-1 (PFOS, acid)	16
45285-51-6 (PFO,ion)	58	45298-90-6 (PFOS, sulfonate)	3

**Table 12:** **Total number of publications about PFOS registered in SciFinder and WoS**

Database	Search term	Publications
SciFinder	Perfluorooctane sulfonate/rt	510
	Perfluorooctane sulfonic acid/rt	173
	45298-90-6/rn	249
	1763-23-1/rn	769
	PFOS/rt	848
	**All**	**1252**
Web of Science	Perfluorooctane sulfonate/tp –all spellings	680
	Perfluorooctane sulfonic acid/tp-all spellings	84
	PFOS/topic	662
	**All**	**685**

**Table 13:** **Number of articles about PFOS in different environmental journals analyzed by WoS and various name forms in SciFinder**

Journal	SciFinder	SciFinder	SciFinder	SciFinder	SciFinder	SciFinder	WoS
	PFOS/rt	45298-90-6/rn	Perfluorooctane sulfonate/rt	1763-23-1/rn	Perfluorooctane sulfonic acid/rt	/all	/all ^1)^
Environmental Science and	124	39	76	89	89	148	150
Technology							
Chemosphere	45	8	23	36	36	52	56
Environmental Toxicology and Chemistry	20	10	15	17	11	28	28
Toxicological Sciences	17	7	15	12	6	21	26
Environmental Health Perspectives	18	6	6	13	13	20	23
Journal of Chromatography A	9	3	4	8	2	16	23
Archives of Environmental Contamination and Toxicology	16	6	9	9	9	17	18
Toxicology	15	6	4	14	14	15	17
Environmental Pollution(Oxf)	12	4	8	8	7	14	14
Reproductive Toxicology	n.i.	n.i.	n.i.	n.i.	n.i	n.i.	13

n.i.= not included.

*1*) All: (PFOS or “perfluorooctane sulfonate” or “perfluoro octane sulfonate” or “perfluorooctanesulfonate” or “perfluorooctane sulfonic acid” or “perfluorooctanesulfonic acid”)/tp.

**Table 14:** **Number of articles about PFOS in different environmental journals with total counts in SciFinder and WoS**

Journal	SciFinder	WoS	SciFinder+WoS	Total
Environmental Toxicology and Chemistry	1	1	27	29
Toxicology	0	2	15	17
Environmental Pollution (Oxf)	0	0	14	14
Toxicological Sciences	0	5	21	26
Journal of Chromatography A	1	8	15	24
Environmental Health Perspectives	0	3	20	23

The SciFinder results using the ‘research topic’ index are obtained with the search terms ‘as entered’. With the search term formulated as a ‘concept’, a major number of apparently erroneous publications with perfluorooctanoate (PFO) or perfluorooctanoic acid (PFOA) are obtained. These substances are part of the larger substance classes perfluorochemicals or perfluorinated acids. Table 11 demonstrates that. We search the term ‘PFOS’ as a ‘concept’ and the result obtained is 2095 publications. If we subtract the 848 publications with PFOS ‘as entered’ it leaves the final result 1247 publications. When these are analyzed by CAS number only few publications are related to PFOS proper.

The totals from all indexed journals (SciFinder and WoS) are shown in Table 12. A number of interesting results can be obtained from this table. Overall, by far, no single search term leads to all publications about PFOS. At most 68% of all publications are found in SciFinder using one single search term. In WoS, the term perfluorooctane sulfonate results in almost all the publications obtained for this substance. Also interesting is the reasonable agreement between articles on perfluorooctane sulfonate in Wos and SciFinder, while this is not the case for perfluorooctane sulfonic acid. The total number of publications about PFOS irrespective of the search method in SciFinder is 1252 articles while the same number in WoS is 685. A significantly larger number of journals are indexed by Scifinder and this database includes 12 percent patents as well.

In Table 13, we present the results for the 10 environmental journals with most articles in WoS. Again, SciFinder gives rather different results whether we search the substance acronym, the chemical name or the unique CAS number. The PFOS acronym or the perfluorooctane sulfonic acid CAS number gives the largest number of articles, but, as demonstrated with the journals investigated, not all relevant articles are included in the simple CAS number search. The results from the similar search in WoS on either PFOS, the sulfonate or sulfonic acid generally lead to more articles.

If we consider registration of individual articles (Table 14) the case of the ‘Journal of Chromatography A’ demonstrates that a search in this journal may lead to eight more articles apparently not included in SciFinder. A close examination of PFOS in these articles reveals that they are actually registered in SciFinder as a salt of the sulfonic acid and not the sulfonate (lithium perfluorooctane sulfonate e.g. with registry number: 29457-72-5). In this way, a registration in the databases considered here depends on whether the substance is represented as an ionized acid or as a salt of this acid. Of note is also the difference in the journal ‘Toxicological Sciences’. Five more articles are registered in WoS compared to Scifinder: In three cases the keyword PFOS are applied in WoS despite the main subject of the article is about perfluoroalkyl acids. This keyword is apparently not accepted by Scifinder. In the last two cases the articles are conference supplements which are not indexed by Scifinder.

The analysis for individual journals demonstrates a fair agreement with the number of articles obtained either with SciFinder or WoS provided that the proper CAS numbers and the different chemical manifestations of the substance are taken into account.

## Discussion

The four different databases we include in the present work seemingly represent different levels of indexing policy with regard to chemical substances. As the leading chemical database we expect SciFinder has the most extensive analysis of chemical content. WoS or Scopus use a more restrictive method while Scholar uses a comprehensive indexing of the full text content of the articles. WoS indexes chemicals mentioned in the title, abstract and keyword fields with no regard to the significance of the chemical to the main subject of the articles. This indexing policy goes for Scholar as well but includes, more unfortunately, secondary material as reference lists. In the case of SciFinder the selection criteria for the chemicals are more focused. The chemical must play a more prominent role with regard to the main subject of the article. This may lead to a neglect of some articles compared to the case for WoS or Scholar. On the other hand, in terms of literature searches, this policy may lead to qualitatively better results with less ‘noise’ produced. If we consider the implementation of a statistical analysis the more automatic approach of WoS and Scholar may sometimes be preferred.

The results shown in Table 1 clearly demonstrates that, even for the well-known and well-studied chemicals, the number of times they are represented in some of the major journals can be very different in WoS and SciFinder. The typical difference is about 30%. Generally, SciFinder finds the most articles but in a few prominent cases WoS has the lead. The case with fluoranthene (Table 1) illustrates that, in many cases, application of a CAS number produces the largest number of articles in SciFinder compared to other databases. On the other hand, the result for chlordane demonstrates that the ‘unique’ CAS number not always leads to the most articles in the literature. Various isomeric forms of the same chemical can exist with separate CAS numbers. A comprehensive literature search must obviously include all forms. The same situation is encountered with PFOS in Table 11.

The relatively few results for Scopus presented here show that there is not a major difference compared with SciFinder and WoS. In most cases the results resemble the data obtained with WoS. This may well be expected due to a similar indexing praxis for the two databases. If we look at the sources of information Scholar clearly has the broadest basis. In principle, they include all types of material. The other three databases use a more narrow selection of journals.

The overall numbers of journals indexed by WoS, around 10000, are slightly higher than those processed by SciFinder. If we consider chemistry related journals alone, the difference in number becomes more prominent and in favor of Scifinder. This may not influence all the results obtained here as we mostly compare the same journals.Generally, we find the largest number of articles to a certain substance (same journal and time period) in Scholar, followed by SciFinder and with fewest in WoS and Scopus. The more careful analysis demonstrates that in a few noteworthy cases more articles are found in WoS compared to SciFinder. The main reason hereto can be: 1. Substances can be excluded as a result of the indexing process in SciFinder (e.g. chemicals registered as intermediates or solvents). 2. In order to become indexed any substance must be described in a significant way. This could e.g. be a new route of synthesis, another value of a physical property or use of the chemical.

The numbers of articles found in Scholar are surprisingly large compared to SciFinder and WoS.Indexing of chemicals which occur in the main text of the article produces a surplus of articles in Scholar compared to the other databases. The chemical name found in the full text or in particular the reference list may be more or less relevant in the context of the literature search. A closer examination of the articles obtained may reveal duplicates. In any case a fair amount of seemingly relevant articles are still obtained which are not included in either WoS or SciFinder.

At the moment one must compare the individual titles in the different search sets and through this analysis obtain the largest possible number of articles about a substance. The comprehensive use of DOIs (Digital Objective Identifiers) for articles in journals can ensure that duplicate records are identified. Download of records including DOIs in reference tools could facilitate identifying and removal of duplicates from different databases as well. More elaborate display formats, e.g. deselecting articles with substances mentioned only in the reference lists or in the full text, could improve searches in Scholar and make them more comparable to the other databases.

In the same way, CAS numbers should be used in a standardized manner throughout the chemical bibliographies. Deleted CAS numbers are listed in SciFinder when searching a substance. In the same manner isomers or other variants of a substance could be presented simultaneously. Chemical structures and alternative chemical names (trade names) should be used when available. Chemical identifiers (InChl or SMILES) are not used in the databases analyzed in the present work. At present they play a role in more specialized chemical databases such as ChemSpider or PubChem. An introduction of these identifiers in the larger bibliographic databases could possibly improve the retrieval of chemical substances.

A full comprehensive search of publications about a substance should, of course, also include more specialized databases as e.g. BIOSIS (biology), COMPENDEX (engineering) or CABA (agriculture). It would be advantageous if this type of search could be performed in clusters of chemical databases at the large database providers. In the same manner, Scholar represents a new type of database which gathers information from general sources and a variety of publications. On the other hand, search precision can become a problem in the more general databases

The results deducted from the tables could possibly be biased by an overall difference in the total number of articles registered in the different databases. In order to estimate any possible impact, Table 15 shows the total counts of articles in the journals ‘Chemosphere’ and ‘Bulletin of Environmental Contamination and Toxicology’ during the period 2000–2009. The data for the latter journal are almost independent of the choice of database. In case of ‘Chemosphere’ the article counts fluctuates somewhat between the databases. Most articles are indexed in Scholar followed by SciFinder. The largest difference for these two databases is observed in 2007 with 11% more articles in Scholar. The representative 10-year difference in ‘Chemosphere’ is 3.2% more articles in Scholar compared to Scopus. We would expect more articles in Scholar as miscellaneous material is included. On the other hand, this difference did not show up in the journal ‘Bulletin of Environmental Contamination and Toxicology’.

**Table 15:** **Total number of publications**^**1**^**in the journals** ‘**Chemosphere**’ **and** ‘**Bulletin of Environmental Contamination and Toxicology**’ **as indexed in the different databases SciFinder**, **WoS**, **Scopus and Scholar**

Chemosphere							
Year	SciFinder^1,2^	WoS^1^	Wos/	Scopus^1^	Scopus/	Scholar	Scholar/
			SciFinder		SciFinder		SciFinder
2000	409	419	1.02	434	1.06	434	1.06
2001	561	595	1.06	512	0.91	603	1.07
2002	608	547	0.90	531	0.87	551	0.91
2003	618	611	0.99	559	0.90	618	1.00
2004	597	653	1.09	646	1.08	661	1.11
2005	743	712	0.96	738	0.99	753	1.01
2006	1057	974	0.92	977	0.92	980	0.93
2007	1040	1131	1.09	1133	1.09	1150	1.11
2008	1107	1066	0.96	1071	1.03	1080	0.97
2009	870	839	0.96	879	1.01	892	1.03
2000-2009	7610	7547	0.99	7480	0.98	7722	1.01
**Bulletin of Environmental Contamination and Toxicology**							
**Year**	**SciFinder**^**1**,**2**^	**WoS**^**1**^	**Wos**/	**Scopus**^**1**^	**Scopus**/	**Scholar**	**Scholar**/
			**SciFinder**		**SciFinder**		**SciFinder**
2000	236	234	0.99	234	0.99	235	1.00
2001	246	246	1.00	246	1.00	245	1.00
2002	254	253	1.00	253	1.00	253	1.00
2003	353	353	1.00	353	1.00	351	0.99
2004	335	332	0.99	332	0.99	332	0.99
2005	339	335	0.99	335	0.99	331	0.98
2006	269	269	1.00	269	1.00	265	0.99
2007	249	248	1.00	281	1.13	244	0.98
2008	234	234	1.00	234	1.00	235	1.00
2009	332	329	0.99	329	0.99	335	1.01
2000-2009	2847	2833	1.00	2866	1.01	2830	0.99

The period is 2000-2009.

*1*) Publications include articles, reviews and proceedings but do not include letters, editorials, news, biographical items, corrections and otherwise miscellaneous material.

*2*) Data from CAPLUS only.

The shear amount of new chemicals and the corresponding growth in scientific literature may also warrant the more automatized indexing methods. This could lead to fewer registrations of chemicals in the databases. In order to investigate this possibility, we extract from SciFinder the 100 most published CAS numbers (Top-100) during the period 2000–2009 in the two journals ‘Chemosphere’ and ‘Environmental Science and Technology’. The percentages of articles which deal with the top-100 CAS numbers are listed for each year in Table 16. For both journals there is a weak increase in the registration rate during the period. This seems to indicate an even more thorough indexing practice with regard to chemicals in SciFinder. In WoS this practice is also most likely improved by adding keywords to the database. The data for Tables 15 and 16 with a total number of articles as well as top-100 chemicals demonstrates that the indexing practice in the databases may influence the search results.

**Table 16:** **SciFinder registration praxis**^**1**^

	Chemosphere			Environmental science and technology		
Year	Total number of publications	Publications with a top-100 CAS-nr	%	Total number of Publications	Publications with a top-100 CAS-nr	%
2000	463	288	62.2	1147	598	52.1
2001	608	398	65.5	1259	637	50.6
2002	611	401	65.6	1100	645	58.6
2003	626	397	63.4	1328	711	53.4
2004	607	397	65.4	1355	752	55.5
2005	752	504	67.0	1695	956	56.4
2006	1061	679	64.0	1475	759	51.5
2007	1050	685	65.2	1886	1048	55.6
2008	1111	761	68.5	1712	1095	63.4
2009	874	594	68.0	2211	1439	65.1

*1*) The 100 most registered CAS numbers in the two journals ‘Chemosphere’ and ‘Environmental Science and Technology’ during the years 2000-2009. The list summarizes the number of publications with at least one top-100 chemical substance. The data are analyzed for each year in percentage of the total number of publications published that year. Data from SciFinder.

## Conclusion

We have investigated the registration of a number of environmentally relevant chemicals in four major bibliographic databases used by the scientific community. SciFinder represents a major chemical database. WoS and Scopus are well known for citation indexing but can be used as general, bibliographic bases while Scholar represents an upcoming, subscription free bibliographic database. Our analysis is mostly based on straightforward counting of publications. In order to avoid any bias from different selection of journals, we chose to investigate and compare within the same portfolio in the different databases.

We only performed a random check with rather few chemicals but demonstrate that proper chemical knowledge as well as familiarity with indexing practices improves the search results significantly.In many cases it is necessary to scrutinize the substances under consideration individually for different trivial names, technical names, CAS numbers or isomers. Also, knowledge of the structure or mixtures involving the chemical at hand may lead to improved search results.The CAS number of a substance is a great advantage in most cases. Our study has found some notable exceptions. As an example, the CAS number of chlordane from the Stockholm list produces almost no articles in SciFinder although a fair number of articles in Scopus. Instead, SciFinder discriminates between different isomeric forms as well as ‘technical’ chlordane. All instances leads to a different number of articles. A complete result is only obtained by combining the different searches. The use of acronyms for substances also makes exhaustive literature searches difficult. In some cases substances are only registered under the acronym or refer to different isomeric forms. The former is observed with the substance DES while the latter is demonstrated with PFOS as an example.

The different indexing methods used by the databases might also qualitatively explain the deviating count numbers. SciFinder in particular judges the relevance of a substance for inclusion in the database. The three other databases apply more automatic methods.

The Google like search screen in SciFinder, ‘research topic’, normally produces two sets of results with the search term ‘as entered’ or as a ‘concept’. Normally, the latter, where truncation or alternative spelling is allowed, produces the largest set of relevant publications. We demonstrate, with PFOS as an example, that, from a chemical point of view, erroneous articles can be included which only deal with related substances. In any case, care must be exercised when dealing with the concept ‘research topic’ in SciFinder.

In the one example with glyphosate registered in Scholar, this database includes almost all articles. Also the chemicals from the Stockholm list are referenced much more frequently in Scholar compared to the other databases. A large number of these articles may be discarded for statistical analysis because the substances are only mentioned in an inferior context within the full text or the cited references of the articles.

The present analysis compares search results (sets) within different databases obtained with identical search profiles. We clearly demonstrate, when we compare individual articles (Tables 9, 10 and 14), that a small number of articles found in a minor search result in one database are not always fully included as part of the major search result in another database. Apparently, each database has the possibility of unique articles either not found or indexed by other means in similar databases. In this way, a complete count of articles which refer to individual substances can be exceedingly difficult and tedious to perform.

We investigated the reliability of the overall indexing in two prominent journals (Table 15) during the centennial. The data from ‘Bulletin of Environmental Contamination and Toxicology’ proves that for genuine articles almost the same numbers are registered in the four databases. Unfortunately, the data for ‘Chemosphere’ show deviations of up to around 10%. This factor also has to be taken into consideration when we estimate numbers. Examples with data from more journals may further quantify the effect.

A possible drift in registration of chemicals was further analyzed in SciFinder (Table 16). The result for the top-100 most registered CAS numbers in SciFinder shows a weak increasing tendency.

No single database records all publications about a single substance although Google Scholar almost hit the mark. However, the database uses full text indexing as well as indexing of references, which makes it more difficult to select the most relevant publications. Searching literature about chemical substances has clearly undergone a revolution in the electronic bibliographies but has also left new challenges. The present work demonstrates that straightforward analysis regarding the frequency of occurrence of chemicals can be performed in the four major, bibliographic databases investigated. Still, basic chemical knowledge about the substances and their registration is a valuable prerequisite when performing such searches.

## Authors’ information

Ole Ellegaard has a master in chemistry and PhD in physics. At present he is a subject specialist at the University Library of Southern Denmark. He has published in the fields of laser physics, vaporization, sputtering a.o. Johan Wallin, also at the University Library of Southern Denmark, is a physician and has published in the fields of osteone analysis, bibliometrics a.o.
